# 

**Published:** 1894-06-30

**Authors:** 


**PRICE TWOPENCE.**

No. 405, Vol. XVI.—June 30th, 1894.

[Registered at the General Post Office as a Newspaper for transmission in the United Kingdom and Abroad.]

WITH EXTRA NURSING SUPPLEMENT.
**Per Annum, 10s. 6d., post free.**
**Monthly Parts, 6d.; post free, 8d.**
**Ditto per Annum, 8s. 6d., post free.**

LIBRARY Index S. G. O.

### THE HOSPITAL

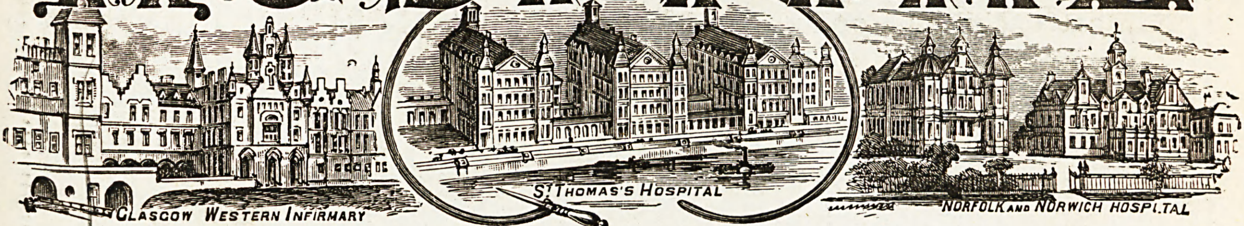

No. 405. Vol. XVI. **WITH EXTRA NURSING SUPPLEMENT.** SATURDAY, JUNE 30TH, 1894.

